# *Protoparvovirus* Interactions with the Cellular DNA Damage Response

**DOI:** 10.3390/v9110323

**Published:** 2017-10-31

**Authors:** Kinjal Majumder, Igor Etingov, David J. Pintel

**Affiliations:** Department of Molecular Microbiology and Immunology, University of Missouri School of Medicine, Bond Life Sciences Center, Columbia, MO 65211, USA; km3k5@missouri.edu (K.M.); etingovi@missouri.edu (I.E.)

**Keywords:** MVM, DNA damage response, cell cycle

## Abstract

*Protoparvoviruses* are simple single-stranded DNA viruses that infect many animal species. The protoparvovirus minute virus of mice (MVM) infects murine and transformed human cells provoking a sustained DNA damage response (DDR). This DDR is dependent on signaling by the ATM kinase and leads to a prolonged pre-mitotic cell cycle block that features the inactivation of ATR-kinase mediated signaling, proteasome-targeted degradation of p21, and inhibition of cyclin B1 expression. This review explores how protoparvoviruses, and specifically MVM, co-opt the common mechanisms regulating the DDR and cell cycle progression in order to prepare the host nuclear environment for productive infection.

## 1. General Overview of MVM-Induced DDR

Upon infecting host cells, DNA viruses provoke DNA damage responses (DDRs), either as a reaction to virally encoded proteins, incoming genomes, or to the large amounts or types of foreign DNA produced during viral replication [1,2]. The cellular DDR is primarily orchestrated by a series of post-transcriptional modifications, which lead to the accumulation of checkpoint, DNA repair, and other effector proteins in the vicinity of DNA lesions. At the core of this virus-induced DDR are a number of well-conserved phosphatidylinositol 3 kinase-related kinases (PIKKs) which coordinate the many arms of the DDR following their rapid redistribution to damaged genomic sites [3]. Upon localization, they phosphorylate cellular targets including local chromatin components in order to label, and subsequently repair, these DNA lesions. The three major PIKKs which control DDR signaling are ataxia telangiectasia mutated (ATM), ataxia telangiectasia and Rad-3 related (ATR), and DNA dependent protein kinase (DNA PK). These kinases induce cellular responses that are varied, and in addition to protecting the genome from physical insult, have the potential to impede or facilitate virus replication. For example, the DDR mobilized in response to adenovirus infection presents a barrier that must be overcome by the virus in order for its replication to proceed [4,5,6,7,8]. This aspect of cellular defense discriminates between the cellular and viral genomes, reflecting the complexity and sophistication of the virus-induced DDR [9]. Herpesviruses, on the other hand, show more complex interactions with the DDR pathway; their replication is attenuated in the absence of some DNA damage proteins [10], whereas the accumulation of certain repair factors at sites of DNA damage are inhibited by the viral immediate early protein ICP0 [10]. In contrast, small DNA tumor viruses such as polyomavirus and SV40 activate a DDR that facilitates their replication [11,12,13]. Following infection of a variety of both murine and human cell types, the parvovirus minute virus of mice (MVM) induces a cellular DDR, which facilitates viral replication [14,15].

Parvoviruses are small non-enveloped icosahedral viruses that are important pathogens in many animal species including humans. MVM is an autonomously replicating parvovirus, a member of the *Protoparvovirus* genus, which is lytic in murine cells and transformed cells of many species. The viral genome is approximately 5 kilobases long, with inverted terminal repeats at each end which form different hairpin structures and serve as origins of replication [16]. The prototype strain MVM(p) is not detectably pathogenic in adult mice. Infection in perinatal mice is inapparent but can be detected by seroconversion [17]. MVM encodes two non-structural proteins: the larger non-structural phosphoprotein NS1 is required for viral replication and expression of the viral capsid gene promoter, while NS2 plays important, currently undefined, roles in the normal murine host, but is dispensable for replication in many transformed cell lines of other species [16]. NS1 binds asymmetrically as a dimer to ACCAACCA octameric consensus sequences at the replication origins where it forms a nicking complex, thereby enabling MVM replication [16]. NS1 also binds to multiple sites on the MVM genome that do not serve as replication origins. NS1 binding near the viral capsid gene promoter P38 is essential for P38 transcriptional activity [16], while it has been suggested that additional binding may have other, as yet undefined, roles in the viral life cycle [18]. Parvoviruses are the only known viruses of vertebrates that contain single-stranded linear DNA genomes, and thus, they present novel replicative DNA structures to cells during infection.

Unlike the DNA tumor viruses, parvoviruses do not drive quiescent cells into S-phase. However, following S-phase entry, cellular DNA polymerase, presumably DNA pol δ, converts the single stranded viral DNA genome into a double stranded molecule that serves as a template for transcription of the viral genes [16]. Upon expression, the NS1 protein interacts specifically with the viral genome to process its replication intermediates. Parvoviruses establish replication factories in the nucleus (termed Autonomous Parvovirus-Associated Replication, or APAR, bodies) where active transcription of viral genes and viral replication takes place [19,20,21]. A classification scheme of APAR bodies based on the morphology of NS1 domains in the nucleus of infected cells has been proposed [15,22]. This system categorizes APAR bodies from Class 0 (small-sized bodies observed three hours into S-phase) to Class IV (very large pan-nuclear structures observed 24 h post infection) [22]. Growth in the size of APAR bodies correlates with the temporal increase in the number of MVM genomes and NS1 expression at these sites as the virus amplifies. Importantly, Ruiz et al. discovered that parts of the cellular DNA repair machinery such as MDC1, recruited to regions of DNA damage by modifications of phosphorylated H2AX (hereafter referred to as γH2AX), form distinct foci at the periphery of APAR bodies [22]. Comet assays, which detect either double-strand breaks, or nicks to cellular DNA, have shown that MVM (Fuller, Etingov, and Pintel, unpublished), as well as other parvoviruses [23,24], cause direct cellular DNA damage. However, the specific cause of cellular DNA breaks, be it replication stress, nicking by NS1, cell cycle arrest, or the induction of reactive oxygen species caused by apoptotic pathways, remains unknown. Together these findings suggest that MVM replication results in localized cellular DNA breaks, leading to the induction of distinct DDR foci around APAR bodies.

**Figure 1:**
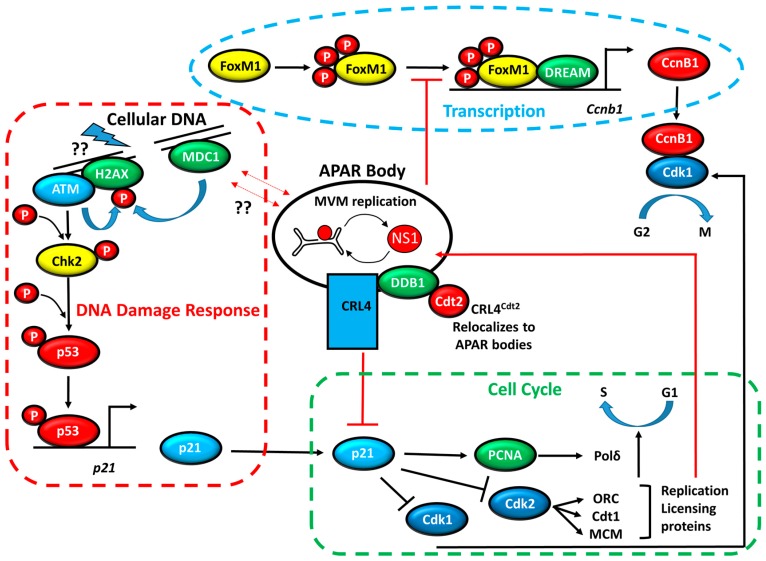
MVM infection integrates signals through the DNA-damage, cell cycle, and transcription pathways. MVM expression and replication occurs in distinct sub-nuclear foci, termed Autonomous Parvovirus-Associated Replication (or APAR) bodies. Cytologically defined by NS1 staining, APAR bodies are nuclear sites where the CRL4^Cdt2^ ubiquitin ligase targets p21 for proteasomal degradation. In addition, APAR bodies serve as depots for the recruitment of cellular DDR machinery. Either binding directly to the viral genome, possibly in response to cellular DNA breaks in their vicinity, or directly to cellular sites of damage, these include, but are not limited to, MRE11, NBS1, ATM, RPA, ATR, DNA-PK, KU70/80, Cdt1, MCM, and ORC [14,27,28]. However, the origins of cellular DNA breaks, and how APAR bodies might associate with broken cellular DNA, remain unknown. Black arrows represent processes that occur in normal cells and red arrows represent processes that take place during MVM infection, including the localization of replication licensing machinery to APAR bodies, inhibition of p21 by CRL4^Cdt2^, inhibition of FOXM1 activation, and the induction of cellular DNA breaks. At early-intermediate stages of infection, these events take place inside of—or in proximity to—MVM-APAR bodies.

MVM replication induces, and is required for, the cellular DDR that helps prepare the nuclear environment for effective parvovirus takeover. Ultimately, the recruitment of DDR proteins depends upon their recognition of a unique signal identifying DNA breaks, in the form of γH2AX, which forms molecular platforms at DSBs [3]. Using specific kinase inhibitors it has been shown that ATM is the primary kinase required for MVM infection-induced DDR signaling [14,22]. ATM phosphorylation of γH2AX would be expected to mark both cellular and viral chromatin that can serve as a platform for MDC1 recruitment to both the viral and cellular genomes [22]. MDC1 can then recruit downstream proteins in the DDR pathway, including the MRN (MRE11-RAD50-NBS1) complex. Similar to SV40 [13], however, MRE11 levels are reduced as MVM infection progresses. This reduction is reversed by treatment with the proteasome inhibitor MG132, suggesting that MVM infection induces the proteasomal degradation of MRE11. Levels of the other components of the MRN complex, RAD50 and NBS1, remain unchanged during MVM infection. This indicates that either there is a special role for MRE11, or that RAD50 plays an MRN-independent role, during MVM infection [14]. Interestingly, recent studies have shown that NBS1 can serve as a substrate for ATM binding at sites of DNA damage, thereby helping to amplify the γH2AX signal [25,26], which may favor the latter of these two possibilities. ATM signaling in uninfected cells activates checkpoint signaling by phosphorylating the kinase CHK2, which subsequently activates the protein p53, and finally, p21, the regulator of a critical G1-S phase checkpoint (Figure 1). However, as discussed below, p21 levels are substantially depleted during MVM infection. Although both MVM and cellular DNA interact with DDR signaling and repair proteins during infection, it is not yet clear in which order these events are executed or how they are associated in space within the nucleus.

During adenovirus infection, viral DNA is first recognized by the MRN complex. MRE11 is subsequently targeted for degradation by viral proteins, and γH2AX then generates a signal that distinguishes cellular from viral DDR [9]. However, the genetic capacity of MVM is much smaller than adenovirus, and it has evolved to induce and co-opt the cellular DDR [14]. As described more fully below, it is noteworthy that MVM-mediated adaptation of the cellular DDR leads to both activation of ATM-mediated signaling and suppression of the downstream components of the ATR signaling pathway. Following MVM infection, cellular genome replication soon ceases while viral replication continues for extended periods of time while the cells are arrested prior to mitosis. Chemical inhibitors of ATM restrict MVM replication and ameliorate the virus-induced cell cycle arrest [14,22]. Thus, MVM exploits the cellular DNA damage response, and the resulting perturbations to the cell cycle, in order to enhance its replication in host cells.

## 2. MVM-Induced Perturbations of the Cell Cycle

Cell cycle dysregulation is one of the cellular responses to DNA-damage signals typically induced by replication stress, genome rearrangements, and external irradiation. Changes in cell cycle regulation can be imposed at the transcriptional, translational, or post-translational levels. This connection between the DDR and cell cycle ensures that the host cell has adequate time and opportunity to repair DNA breaks, thereby preventing mutations from being propagated to daughter cells [29]. In normal cycling cells, the transition from G2 to mitotic phase is governed by activity of the CDK1 (also called cdc2) kinase in the complex with its mitotic cyclin B1 [30]. In the absence of DNA damage, cells depend on Wee1 kinase to phosphorylate CDK1 on Tyr 15, which renders it inactive. When cells cycle to the G2/M border, it is primarily the CDC25C phosphatase that removes this inhibitory phosphorylation, thus promoting the activation of CDK1 and mitotic entry [31]. 

Virus-induced regulation of the cell cycle is a common consequence of infection, and at least in some cases, in response to virus-induced DDR. For example, HIV VPR inhibits the activation of the cyclin B1/CDK1 complex [32,33,34], likely by binding and altering the activity of the CDC25C phosphatase, which is required to remove the inhibitory phosphorylation of CDK1 (described below). Similarly, Human papilloma virus type 1, reoviruses, and SV40 also prevent activation of CDK1 by maintaining it in its inhibitory phosphorylated state [35,36,37]. MVM infection also results in an extended pre-mitotic cell cycle block during which viral replication proceeds, but accomplishes this by a different mechanism, as described below.

During host cell infection, MVM induces a vigorous DDR in murine cells in which p53 is continually activated. Surprisingly, however, p21^WAF1/Cip1^ (hereafter referred to as p21) and CHK1 (as discussed more fully below), major effectors typically associated with S-phase and G2-phase cell cycle arrest in response to diverse DNA damage stimuli, remain depleted or are inactivated, respectively [27,28,38]. Cycling cells typically reversibly down regulate p21 upon S-phase entry or in response to DNA double strand breaks, which is then restored to basal levels upon S-phase exit. Surprisingly, however, this recovery of p21 is not seen following MVM infection, suggesting that MVM dysregulates the p21 activation pathway during the late stages of infection [38]. One role of p21 during the cell cycle is to inhibit CDK1, a kinase that associates with cyclin B1 to modulate G2-M phase transition (Figure 1). Despite the absence of p21 during MVM infection, the host cell fails to transition into mitosis. In contrast to the cellular response to genotoxic agents, in which the DDR halts the cell cycle which then resumes following repair, MVM genome replication provides an ongoing source of sustained DDR, which may require the existence of an atypical mechanism for MVM-induced cell cycle block. These findings suggested that MVM uses a novel strategy to ensure a pseudo S-phase, pre-mitotic, nuclear environment for sustained viral replication.

Experiments designed to monitor cell cycle progression following infection confirm that the G2/M block imposed by MVM prevents the entry of infected cells into mitosis [39]. Normally, the entry into mitosis is tightly regulated and requires the cyclin B1/CDK1 complex to reach threshold levels of activity. Importantly, the kinase activity of the cyclin B1/CDK1 complex in MVM infected cells is significantly reduced, suggesting that the loss of this activity plays a key role in maintaining the pre-mitotic cell cycle block during MVM infection.

Surprisingly, however, during MVM infection, although activity of the cyclin B1/CDK1 complex is lost, the inhibitory phosphorylation of CDK1 at Tyr-15 is absent-typically an indicator associated with activity of the cyclin B1/CDK1 complex [39]. Rather, cyclin B1 and its encoding RNA become substantially depleted during infection. In doing so, MVM prevents cyclin B1/CDK1 activity, thus affecting this critical pre-mitotic checkpoint. In comparison with MVM infection, doxorubicin-mediated arrest of cells at the G2/M border significantly elevates cyclin B1 protein levels [40]. These results affirm that the loss of cyclin B1 observed following MVM infection is a specific, virally-induced event, and likely the cause rather than the consequence of the cell cycle block. 

Studies investigating the production of nascent cyclin B1 RNA revealed that MVM infection specifically reduces the expression of the cyclin B1 gene (*Ccnb1*) [40]. The expression of *Ccnb1* is transcriptionally-regulated in a complex manner during cell cycle progression [31,41]. The *Ccnb1* locus contains nine introns and at least five transcriptional promoter elements regulating its expression, including CCAAT-boxes, GC-boxes, E-boxes, p53 responsive elements, and the cell cycle gene homology region (CHR) [42]. These transcriptional control elements respectively bind NF-Y, SP1, MYC, p53, and the MYB-MUVB (MMB) complex. The CHR and its protein-binding partners cooperatively modulate *Ccnb1* expression [42]. In addition to *Ccnb1*, CHR sites regulate other cell cycle genes including *Ccnb2*, *Cdkd1*, *Cdc25*, and Polo-like kinase-1 (*Plk1*). CHR-regulated genes are typically active during the G2 and M phases, while being repressed during other stages of the cell cycle. The DREAM complex (made up of p130, p107 Dimerization Partners, Retinoblastoma-like, E2F, and MUVB proteins) both represses the transcriptional activity of these CHR genes during G0 and G1, and helps activate them as cells transitioning from late G1 phase into S and progressing towards the mitotic border [43]. As cells progress through late S and into G2/M, the DREAM complex dissociates from cellular promoters, and MUVB remains and successively recruits both B-MYB (forming the MMB complex) and the forkhead transcription factor FOXM1. Proper function of FOXM1 requires the phosphorylation of five to seven sites that reside within its C-terminal transactivation domain. These phosphorylation events, some of which depend upon the kinase activity of the CCNA/CDK complex, are thought to activate FOXM1 by relieving auto-repression caused by the interaction of the C-terminus with an N-terminal repressive domain. 

Studies have shown that diminished transcription of the *Ccnb1* gene in MVM-infected cells is caused by a significantly-reduced occupancy of RNA polymerase II (RNA pol II) on the *Ccnb1* promoter, thus implicating the role of chromatin accessibility mediated by transcription factors at the *Ccnb1* locus. Consistent with this hypothesis, a significant reduction in the binding of FOXM1, and its phosphorylation (likely active form), at the *Ccnb1* promoter during MVM infection has been observed (Figure 1, [40]). Gain of function experiments in which a constitutively active FOXM1 or the FOXM1 transactivation domain was targeted to the *Ccnb1* promoter, using dead-CAS9-FOXM1 fusion proteins in the presence of *Ccnb1* promoter guide RNAs, rescued both *Ccnb1* RNA and protein expression in infected cells [40]. These results implicate FOXM1 as a critical target for *Ccnb1* inhibition by MVM, which contributes to the pre-mitotic block evoked during viral infection. *Plk1*, one of the cell cycle genes regulated by CHR sites (described above), is the primary kinase that phosphorylates (and thereby activates) FOXM1, which otherwise enables cells to transition into mitosis [44]. This mechanism of autoregulation of cell-cycle regulated genes may serve as a target for MVM-mediated transcriptional repression.

The dysregulation of *Ccnb1* may reflect a common mechanism shared by many viruses in their attempts to exploit the cellular cell-cycle machinery. HIV can also utilize its Tat protein to stimulate the expression of *Ccnb1*, which is thought to promote apoptosis, and then targets cyclin B1 for proteasomal degradation by binding to its N-terminus [45]. The E4 protein of HPV16 is able to sequester the cyclin B1/CDK1 complex to the cytoplasm, thus preventing its nuclear localization and activity [46,47]. The expression of the *Ccnb1* gene can also be regulated at the level of its RNA stability. Interactions of the cellular RNA binding protein HuR within the 3^′^ UTR have been shown to affect the stability of *Ccnb1* RNA under certain conditions [48]. As described, MVM uses a very different approach to prevent the activation of the cyclin B1/CDK1 complex: infection inhibits the production of *Ccnb1* RNA which leads to the loss of cyclin B1 protein. It has been noted that expression of the NS1 protein from the related rat parvovirus H-1PV leads to G2-phase arrest, which essentially phenocopies the cell cycle checkpoint observed in H-1PV infected cells [49]. Surprisingly, however, ectopic expression of the MVM-NS1 protein alone leads to an increase, rather than decrease, in cyclin B1 levels. This suggests that a unique interaction with the cell cycle machinery during infection mediated by viral replication and the DDR triggers *Ccnb1* inhibition [40].

## 3. Regulation of p21 by MVM Infection

p21 typically plays an important role in S- and G2-phase cell cycle arrest in response to diverse DNA damage stimuli [50]. p21 is a CDK1 inhibitor which helps maintain the cyclin B1/CDK1 complex in an inactive state, thereby preventing mitotic progression (described above). The crucial role of p21 in regulating the cell cycle has rendered it a susceptible target for viruses that seek to influence the cell cycle machinery. For example, HIV VPR upregulates p21 to inhibit cyclin B1/CDK1 activation [51,52]. However, as mentioned above, p21 levels are substantially depleted during MVM infection. This compels MVM to utilize other mechanisms to halt cell cycle progression, but also affords an important advantage to the replication of MVM.

During MVM infection, p53, a transcriptional activator of p21, is significantly up-regulated and activated throughout MVM infection. Remarkably, however, while this does lead to the expression of p21 mRNA, p21 protein levels remain low throughout infection, including during the prolonged pre-mitotic phase in which the viral genome is replicated [38]. Depleted levels of p21 can be restored by treating infected cells with the proteasome inhibitor MG132, suggesting that p21 degradation during MVM infection is performed by the proteasomal machinery. Interestingly, however, whereas ATR activity modulates p21 degradation in response to various DDR-inducing agents (as described more fully below), the activation of ATR and its substrate CHK1 is reduced during MVM infection, consistent with the independence of p21 signaling from this pathway [28]. Similar to the case described above for cyclin B1 expression, while MVM infection leads to a loss of p21, the ectopic expression of NS1 results in increased p21 levels [39]. Together these results suggested that a novel mechanism depletes p21 levels during MVM infection, and continued degradation of p21 was likely necessary for efficient virus replication.

MVM-induced depletion of p21 is an efficient mechanism to maximize MVM replication in its host cell. As a key cell cycle and DNA synthesis regulator, p21 has been shown to be an effective inhibitor of MVM replication via its interaction with PCNA [53], an important co-factor for DNA polymerase δ which is thought to replicate the MVM genome.

MVM replication requires the re-localization of the E3 ubiquitin ligase CRL4^Cdt2^ to MVM APAR bodies, where it targets p21 for degradation (Figure 1). This recruitment is specific for the CRL4^Cdt2^ ligase because the APC/C^Cdc20^ E3 ligase, which targets p21 for degradation after mitotic entry, is not similarly recruited [27]. The Cullin-RING Ligase (CRL) CRL4^Cdt2^ possesses unique properties that enable it to couple DNA synthesis with proteolysis. It is made up of a scaffold protein Cullin 4 and a homo-trimeric protein DDB1, which serves as an adaptor for the putative substrate recognition protein Cdt2 [54,55,56]. CRL4^Cdt2^ has been shown to program the ubiquitination and subsequent degradation of p21 in response to DNA damaging agents such as UV treatment in order to ensure low p21 levels during S-phase [54,56]. Upon DNA damage or S-phase entry, CRL4^Cdt2^ is recruited to chromatin via interaction with the DNA pol δ co-factor PCNA where it targets substrate proteins for degradation [57]. PCNA provides a molecular platform for substrate recognition by the CRL4^Cdt2^ E3 Ub ligase, leading to the targeting of p21 for proteolytic degradation. p21 binds to PCNA via its PCNA-Interacting Protein (PIP) box, a conserved motif shared by substrates of the CRL4^Cdt2^ ligase [27].

Both PCNA and DNA polymerase δ are localized to APAR bodies [27]. By assessing viral replication in permissive cells that were engineered to inducibly express wild-type or mutant p21, it was shown that p21 depletion is necessary to specifically prevent its inhibitory interaction with PCNA and thus replicative activity of DNA polymerase δ. Expression of a stable p21 mutant that retains its interaction with PCNA inhibited MVM replication, whereas a stable p21 mutant which lacked this interaction did not. Additionally, the introduction of a p21-derived peptide attached to a penetratin motif that specifically prevented p21-PCNA interaction also substantially decreased viral replication [27]. Taken together, these findings suggested that while interaction with PCNA is important first for targeting p21 to the CRL4^Cdt2^ ligase (re-localized to MVM APAR bodies), efficient viral replication requires the subsequent depletion of p21 to abrogate its inhibition of PCNA. p21 also inhibits the function of CDK2, a cellular kinase activated in early S phase that helps induce the licensing machinery for DNA replication. The methyl-transferase SET8 and the replication licensing factor Cdt1 (Cdc10-dependent transcript 1; no structural relationship to Cdt2) are also well-characterized substrates of the CRL4^Cdt2^ E3 Ub ligase. Similar to p21, SET8 is degraded during MVM infection; however, surprisingly, Cdt1 remains stable although localized to APAR bodies [27]. Therefore, by specifically regulating cellular p21 levels, MVM can take control of the licensing and replication machinery from the cell to carry out synthesis of the viral DNA.

## 4. Suppression of CHK1 Activation by MVM Infection

ATM, ATR, and DNA PK are the major kinases that coordinate the DNA damage response to diverse DNA damage stimuli [3]. Unlike ATM, ATR is essential for cellular survival, and it has essential functions in the maintenance of genome integrity in response to replication stress in S- and G2-phases [3,58]. As mentioned above, ATR activation of CHK1, a major effector typically associated with S-phase and G2-phase cell cycle arrest in response to diverse DNA damage stimuli, is inhibited during MVM infection [28].

The regulation of CHK1 activation and its ensuing effect on DDR, as well as the cell cycle, is complex and new data is continuing to emerge. Briefly, the CHK1 Ser/Thr protein kinase is comprised of N-terminus catalytic and C-terminus regulatory domains. In the course of progression through the unperturbed cell cycle, CHK1 maintains a folded conformation by virtue of association between its kinase and regulatory domains. The folded protein is associated with nuclear chromatin, and thus CHK1 kinase activity is very low in both the nucleoplasm and cytoplasm in the absence of DNA damage [59]. Interestingly, the chromatin-bound kinase is reported to have local activity, phosphorylating histone H3 at Thr11. This leads to the acetylation of H3 at Lys9, resulting in a significant increase in the transcription levels of several genes during normal cell cycle progression [60].

Following mutagenic activity, ATR is recruited to RPA-coated single-stranded DNA at DNA damage sites via its interacting partner ATRIP, which binds to the large subunit of RPA. In a separate recruitment event, the chromatin protein RAD17 loads the heterotrimeric ring shaped 9-1-1 complex (which consists of RAD9, HUS1, and RAD1, and resembles the replication sliding clamp) onto free 5^′^ termini at stalled replication forks and recessed DNA ends. Full activation of ATR requires TOPBP1, which is recruited to DNA lesions via the 9-1-1 complex [3,58]. The RAD17/9-1-1 complex also interacts with claspin, a CHK1 binding partner that recruits CHK1 to ATR. Phosphorylation of CHK1 at residue Serine 317 (S317) and Serine 345 (S345) by activated ATR results in disruption of the association between the enzymatic and regulatory CHK1 domains (described above), followed by release from chromatin of the active enzyme into the nucleoplasm and later to cytoplasm. The released CHK1 in turn phosphorylates several targets such as WEE1, CDC25A/B/C, and others, leading to a number of cellular effects including, among others, G2/S checkpoint activation, the inhibition of new replication-origin firing, and stabilization of replication forks [59]. At the same time, the dissociation of CHK1 from chromatin has been shown to decrease levels of H3 Thr11 phosphorylation/Lys9 acetylation and to significantly lower transcription levels of genes known to be suppressed during DDR [60].

Thus, ATR activities are mediated in large part by CHK1, and as a result, the phosphorylation status of CHK1 is often employed as a surrogate for ATR activation. An independent mechanism of ATR activation is carried out by the RPA-binding protein ETAA1 (Ewing’s Tumor Associated Antigen 1). Similar to TOPBP1, ETAA1 harbors AAD domains, providing host cells with a 9-1-1/TOPBP1-independent axis for ATR activation, thereby providing a redundant mechanism to respond to replication stress [61].

The progression of MVM replication in APAR bodies generates substantial amounts of RPA-coated single-strand DNA [14,62]. Together with a number of other DNA repair proteins, both ATR and its associated protein ATRIP are recruited to APAR bodies during the replication of MVM. Surprisingly, however, CHK1 is not activated post-infection when single-stranded viral genomes bear bound RPA. RPA is normally a potent trigger of ATR activation, and is present in APAR bodies [28]. Single-strand nicks, which are normally another trigger of ATR activation, are generated during MVM infection and may be occluded by bound NS1. In addition, caffeine (which inhibits both ATM and ATR) does not inhibit MVM replication significantly greater than an ATM inhibitor alone [14].

Failure to activate CHK1 in response to MVM infection is not due to the degradation of specific components of the ATR signaling pathway, but rather is consistent with the observation that RAD9, and TOPBP1, which requires the 9-1-1 complex for targeting, failed to associate with MVM chromatin at APAR bodies. Although there is a transient, modest activation of ATR early during infection prior to the onset of the virus-induced DDR, upon the establishment of full viral replication, ATR phosphorylation becomes undetectable, and the activation of CHK1 in response to HU and various other drug treatments is also prevented. Interestingly, the absence of CHK1 phosphorylation at S345 was also reported for HSV-1, although this virus causes a DDR and recruits elements of the ATR pathway to its replication centers. This phosphorylation was suggested to be prevented by the binding of an HSV protein complex to DNA, which prevented association of the 9-1-1 complex with the viral genome and other components of the ATR/CHK1 activation machinery [63].

Additionally, although MVM infection induced RPA32 phosphorylation on serine 33, an ATR-associated phosphorylation site, this phosphorylation event cannot be prevented by verified ATR depletion or drug inhibition [28]. The partial activation of ATR in the early stages could potentially be attributed to ETAA1, which can be recruited to phosphorylated RPA32 and can activate ATR independently of 9-1-1/ TOPBP1 (described above). However, if this is the case, it remains to be determined why ETAA1 does not fully activate the ATR pathway during MVM infection. Even though MVM infection disables ATR signaling during the virally-induced DDR, certain downstream targets typically activated by ATR remain modified.

## 5. Outstanding Questions

Despite a wealth of knowledge about how MVM infects host cells, induces a DDR, and exerts a p21- and CHK1-independent cell cycle arrest, many questions remain about the nature of MVM-DDR interactions during infection. For example: (i) it is still not clear where the sites of cellular DNA damage reside and how MVM communicates with them; (ii) it remains unclear how cellular DNA is damaged by infection; (iii) it is not known how MRE11 is degraded during MVM infection and whether there are MRE11-independent function of NBS1 and RAD50 at APAR bodies; (iv) the mechanism of ATR inactivation during MVM infection is not fully understood, and it is not known if non-phosphorylated CHK1 plays a role during infection; and (v) it is also not known how MVM infection affects cellular gene expression on a global scale.

But perhaps most importantly, in spite of many findings over the past decade addressing how MVM induces a DNA damage response and cell cycle block in its host, we are yet to fully understand the integrative nature of these phenomena between virus and host. The application of –omics-based techniques and high-resolution single-molecular studies over the coming years will yield significant insights into the detailed mechanisms of this essential virus-host interaction.
